# Psychoeducation as an active ingredient for interventions for perinatal depression and anxiety in youth: a mixed-method systematic literature review and lived experience synthesis

**DOI:** 10.1192/bjo.2023.614

**Published:** 2023-12-13

**Authors:** Wezi Mhango, Lucie Crowter, Daniel Michelson, Darya Gaysina

**Affiliations:** School of Psychology, University of Sussex, UK; and Department of Psychology, University of Malawi, Malawi; School of Psychology, University of Sussex, UK

**Keywords:** Psychoeducation, depression, anxiety, adolescence and youth, perinatal interventions

## Abstract

**Background:**

Psychoeducation is a common element in psychological interventions for youth depression and anxiety, but evidence about its use with youth perinatally is limited.

**Aims:**

This review aims to understand outcomes and mechanisms of psychoeducation for the indicated prevention and treatment of perinatal depression and anxiety in youth.

**Method:**

For this review, we synthesised published quantitative and qualitative evidence. Seven databases (ASSIA, Medline, PubMed, PsycINFO, PsycArticles, Scopus and Web of Science) were searched for studies published before 10 August 2021. We also had consultations with a youth advisory group (*N* = 12).

**Results:**

In total, 20 studies met the inclusion criteria. Seven quantitative studies examined multicomponent interventions that included psychoeducation, and one study evaluated psychoeducation as a standalone intervention for postnatal depression. Multicomponent interventions showed significant effects on postnatal depression in two out of six studies, as well as being effective at reducing prenatal anxiety in one study. Standalone psychoeducation for postnatal depression was also effective in one study. Evidence from 12 qualitative studies, corroborated by commentaries from the youth advisory group, suggested that psychoeducation could increase knowledge about symptoms, generate awareness of relevant services and enhance coping.

**Conclusions:**

Psychoeducation may be an important foundational ingredient of interventions for perinatal depression and, potentially, anxiety in adolescents and young adults through stimulating help-seeking and self-care.

## Perinatal depression and anxiety in youth

Young women are especially vulnerable to depression and anxiety in the perinatal period, comprising pregnancy and the first year after childbirth. This is because of the challenges of adjusting to pregnancy and early child-rearing, as well as navigating developmental tasks and psychosocial risk factors that occur during adolescence and early adulthood more generally.^1,2^ Among pregnant and postpartum women aged under 25 years, an estimated 16–26% experience prenatal and postpartum depression (PPD) and/or anxiety, which amounts to approximately 60 million cases per year globally.^3^ This is higher than the prevalence rates for older women, which are estimated at 11.9% for perinatal depression^3^ and 4.1–5.7% for perinatal generalised anxiety disorders.^4^

Research on perinatal mental health has shown that adolescents aged 19 years and younger experience similar challenges with young adults aged 20–24 years.^5,6^ These include disrupted education achievement and participation in employment, relationship stresses, low social support and housing tenure. Although these factors are prevalent in both high- and low-income countries, low education levels and food insecurity have been reported to be more common in low- and middle-income countries (LMICs), as they are closely linked with poverty.^7–9^ However, being a younger mother has been found to be more highly associated with perinatal depression and anxiety, as younger adolescents are often isolated and ridiculed by their peers.^10^ In addition, although the prefrontal cortex (which is responsible for cognitive functioning) develops throughout pregnancy and continues into early adulthood,^11^ younger adolescents may have lower emotional and cognitive abilities for dealing with motherhood alongside navigating their own developmental changes.^12^ Apart from the associated adverse effects on maternal health and functioning, perinatal depression and anxiety can negatively affect child development and perpetuate intergenerational transmission of mental disorders.^13–15^

## Psychoeducation for perinatal mental health interventions

Psychoeducation involves structured communication of information about mental health problems and how these can be improved. It is one of the most used practice elements (i.e. discrete clinical techniques or strategies forming a larger intervention plan)^16^ in perinatal mental health interventions, and in evidence-based psychotherapies more generally.^17^ Psychoeducation can improve mental health by enhancing the capability and motivation of individuals to change behaviours that affect mental health, and by increasing receptiveness to seek help for mental health problems.^18,19^ Psychoeducation incorporates a combination of (a) providing information about mental ill health and its causes, prevention, treatment and ongoing management; (b) skills training to develop behaviours to prevent or alleviate symptoms and functional impairments; and (c) relational elements for enhancing motivation to engage with intervention tasks and apply new learning (Box 1).^19^

Box 1 Common content areas in psychoeducation

- Aetiological factors/causes of the condition
- Common signs and symptoms
- Early signs of relapse or recurrence
- Skills for self-management of symptoms
- Available treatment options
- How and when to seek treatment
- Importance of adherence to treatment
- Long-term course and potential outcomes
- Addressing myths and misconceptions about the condition to reduce stigma

In psychoeducation interventions, the informational component is often conceptualised in terms of promoting ‘mental health literacy’,^20^ whereas the skills-based component may involve structured training in complementary behavioural practices such as relaxation or problem-solving. Psychoeducation can be delivered individually or in groups, through self-directed methods (e.g. use of booklets/pamphlets, videos) or provided through an external facilitator/therapist, and can be adapted to reflect diverse population needs and settings.

Although psychoeducational interventions have been widely used in perinatal interventions targeting depression and anxiety, existing reviews of this approach are quite limited in their scope, thereby making it difficult to draw inferences on their effectiveness. Previous systematic reviews and meta-analyses found that psychoeducational interventions were effective in decreasing symptoms of depression, anxiety and psychological distress in general populations,^21,22^ and promoting maternal mental health in pregnant women ^23^ and among adolescents in the perinatal period more specifically.^24–27^ However, these reviews have been limited in their evidence. There is a scarcity of studies from LMICs, which have the highest rates of youth pregnancy,^28,29^ which limits the generalisability of the results to non-Western countries. In addition, these studies only focused on quantitative studies. To address these gaps, the current review triangulated quantitative evidence, qualitative evidence and commentary from a lived experience panel with participants from diverse backgrounds (primarily LMICs), to increase the credibility and generalisability of the findings.

This review summarises evidence for the use of psychoeducation as an active ingredient for the prevention and treatment of perinatal depression and anxiety in 14- to 24-year-olds (as part of the Wellcome Trust ‘active ingredient’ initiative).^30^ Active ingredients can be defined as elements that can be directly targeted to keep the focus on evaluating tangible solutions as well as acknowledging complexity.^30^ The review sought to address the question, does psychoeducation work and for whom (research question 1)? The review also sought to explore in which settings and contexts psychoeducation works (research question 2). Finally, the possible mechanisms through which psychoeducation may affect perinatal depression and anxiety in youth were explored, answering the question, why does psychoeducation work (research question 3)?

## Method

### Protocol and registration

The study protocol was registered with International Prospective Register of Systematic Reviews (PROSPERO) on 13 September 2021 (identifier CRD42021273877).

### Information sources and search strategy

We conducted a mixed-method evidence synthesis, using (a) a systematic review of quantitative studies that evaluated perinatal depression and/or anxiety outcomes among 14- to 24-year-olds participating in interventions containing psychoeducation; and (b) a thematic meta-synthesis of qualitative evidence on young people's perceptions of utility, acceptability, and barriers and facilitators of using psychoeducation during the perinatal period. Methods and results were reported in accordance with the Preferred Reporting Items for Systematic Reviews and Meta-Analyses (PRISMA) guidelines.^31^

We searched Medline, PubMed, PsycINFO, PsycArticles, ASSIA, Web of Science and Scopus databases, with search terms combined using Boolean operators:

- Intervention (education* OR information OR knowledge OR literacy OR awareness) AND
- Diagnosis (depress* OR anxi* OR ‘mental health’ OR ‘mental disorder’ OR ‘emotional disorder’ OR psychiatric) AND
- Period (perinatal OR antenatal OR prenatal OR postnatal OR postpartum OR pregnan*) AND
- Age (youth OR young OR adolescen* OR teen*) AND
- Context (Intervention OR prevention OR treatment OR manag*).

Additional studies were selected from reference list searches for all included articles derived from the systematic search, and from reference list searches of previous reviews of psychoeducation for young people. All the identified studies were subject to the inclusion and exclusion criteria.

### Inclusion and exclusion criteria

#### Participants/population

The main inclusion criterion was participants’ age at the time of perinatal intervention. Studies focusing on youth aged 14–24 years were eligible. In terms of adolescent and youth mental health studies, 14 years of age is used as the cut-off given that the period from 14 years of age to early adulthood is the key onset period for most mental health problems.^32^ In addition, although the age of consent varies internationally (12–21 years), most countries have the age of consent as 14 years, with fewer countries having the age of consent below 14 years.^33^ This early sexual debut leads to early pregnancies that, in turn, put adolescents at high risk of perinatal mental health problems such as depression and anxiety.^1,2^ However, because of the limited number of studies focusing on the target age range, studies with a wider age range within which the population comprised at least 50% of 14- to 24-year-olds were included. For studies that did not report the age range, those with a mean of 24 years or less were included.

#### Interventions

Interventions containing psychoeducation, defined as structured communication of information intended to increase awareness and understanding of mental health problems and their prevention, management and treatment, were included. Psychoeducation interventions delivered in any form (individual, group, family, peer-led, professional-led) or mode (in-person, telephone, online) were included. Studies were included if psychoeducation was delivered as a standalone or part of a multicomponent intervention.

For qualitative studies, we considered qualitative studies that examined experiences of perinatal interventions containing psychoeducation, barriers and facilitators of psychoeducation, and studies of information needs and/or preferences related to perinatal depression and anxiety in youth.

#### Context

Studies in any geographical location were included. Studies conducted in any health, community or educational setting were included.

#### Outcomes

For quantitative studies, primary outcomes of interest were depression and anxiety during pregnancy or in the first year following delivery. We included studies where depression and/or anxiety were assessed by diagnostic status (based on clinical research criteria indexed against the DSM or ICD classifications), or where symptoms of depression and anxiety were measured with validated self-report instruments and/or screening tools. Qualitative studies were included if they investigated psychoeducation in relation to help-seeking and prevention or treatment of depression and/or anxiety.

#### Additional outcomes

Secondary outcomes of interest were extracted from studies that reported on depression or anxiety. These additional outcomes included mental health literacy, coping mechanisms, and child-related and parenting-related outcomes. Among qualitative studies, we wanted to explore influences on the effectiveness and acceptability of psychoeducation.

#### Study design

Eligible study designs included randomised controlled trials, controlled pre–post interventional studies and studies that collected data from qualitative interviews/focus groups.

### Study selection

Covidence software (see https://www.covidence.org)^34^ was used to manage the screening process. Three reviewers were involved in the process of searching and selecting studies and 10% of all records were double-screened at the abstract, title and full-text screening stages. Disagreements between individual reviewers were resolved through group discussions. Cohen's kappa was used to calculate interrater reliability, and interrater reliability was found to be substantial (0.75).^35^

### Data extraction

Two reviewers independently extracted data, with regular meetings to discuss the process. Data were obtained on the study design, geographical location and delivery setting, sample characteristics, intervention characteristics (psychoeducation as a multicomponent or standalone intervention, delivery format, mode, provider, duration), relevant outcome measures and/or qualitative data collection methods, details of mediation and/or moderation analyses (if applicable). For qualitative studies, extracted data included all information in the results section only. Original authors were not contacted for missing information because of the rapid nature of the review.

### Quality assessment

Two independent reviewers assessed study quality and possible bias with the Mixed Method Assessment Tool (MMAT).^36^ Any discrepancies were resolved through discussion. We did not exclude any studies based on predetermined quality thresholds.

### Consultations with the youth advisory group

As part of patient and public involvement, we recruited an international youth advisory group (YAG) to draw inferences from the published evidence. A digital flier detailing aims of the workshops, the role of young advisors and eligibility criteria (experience of pregnancy or parenthood before the age of 25 years) was circulated through existing research and practice networks. Those interested were asked to contact a researcher (L.C.) via email.

The YAG comprised 12 participants of different nationalities (aged 17–26 years, one male and 11 females) who self-identified as having lived experience of youth pregnancy. Four virtual Zoom consultation meetings consisted of semi-structured discussions on the acceptability and utility of psychoeducation, the credibility of the preliminary evidence synthesis, potential refinements to the synthesis and practical implications. The YAG participants were not involved in the framing of review questions or methods. However, they were involved in the interpretation of findings. For each session, the number of young advisors in attendance varied between six and eight. All discussions were led by members of the research team (L.C., W.M. and D.G.). The YAG commentaries were organised around research questions and were used to guide the iterative synthesis of the evidence sources (see Data synthesis).

The University of Sussex research governance team reviewed the role of the YAG in this project and confirmed that the methods and aims were consistent with guidelines on patient public involvement, rather than constituting primary research.^37^ As such, the project was deemed to be exempt from a formal ethics review. Nevertheless, we obtained verbal consent from all participants to record the YAG meetings and use their anonymised commentaries in written reports and other dissemination material. At the start of every Zoom meeting, we obtained permission from the YAG to record the consent process. Once the participants agreed, a consent form was displayed, and a researcher read through and explained each statement. Participants were asked if they agreed with the statements and whether they wanted to participate in the meeting. If not, participants were free to leave the meeting without any explanation.

### Data synthesis

We followed a meta-ethnographic approach where qualitative evidence was thematically analysed,^38^ and we used the convergent mixed-methods design to integrate qualitative and quantitative results.^39^ A narrative synthesis of evidence was conducted as there was not sufficient data for a meta-analysis. Summaries of interventions, structured around the type of intervention, content, outcomes and population characteristics are presented in tables. The strength of evidence for the effectiveness of psychoeducational interventions, as well as evidence for mediators and moderators of effectiveness, were examined. Putative moderators included those reflecting the geographical location, setting (i.e. primary healthcare, community, educational, clinical), duration and format of interventions, and participant characteristics, including age and perinatal period (prenatal or postnatal). This review was also shaped by members of the YAG who engaged with summaries of the qualitative and quantitative evidence. This enabled them to validate or challenge the interpretations that were drawn by the research team, ensuring that these inferences were relevant to the needs of young people.

## Results

### Description of included studies

**Fig. 1:**
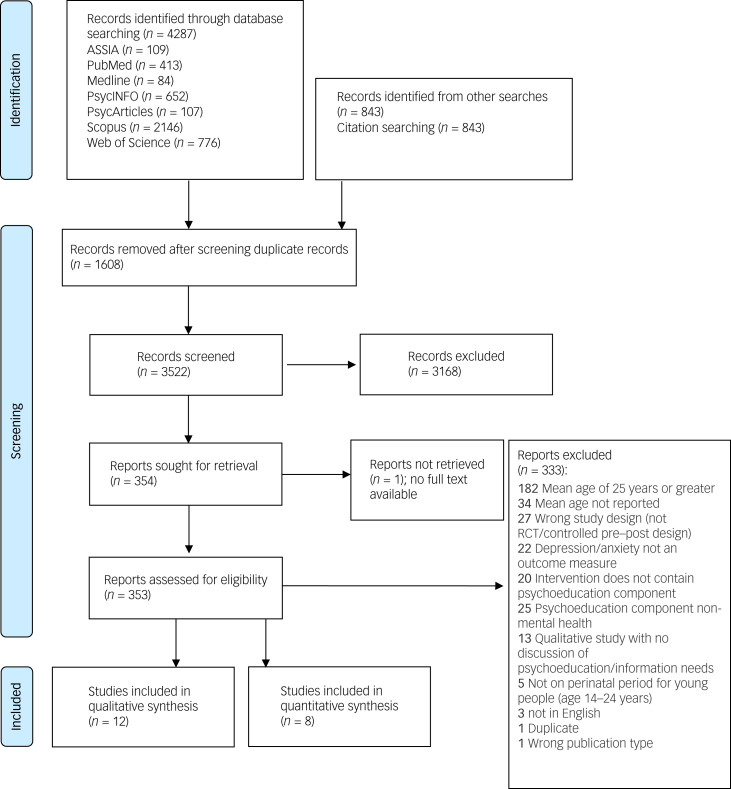
Preferred Reporting Items for Systematic Reviews and Meta-Analyses flow diagram^32^ of the study selection process.

In total, 20 eligible studies were identified and included in the review (Fig. 1). Characteristics of both quantitative and qualitative studies are summarised in Table 1.

**Table 1:** Characteristics of studies included in the systematic literature review

Author (year)	Number of participants	Country	Location	Method	Comparison	Primary outcomes (measures)	Secondary outcomes	Intervention type (eligibility)	Mean age (range)	Modality	Provider	Sessions
**Quantitative studies**												
Bastani et al (2005)^40^	110	UMIC (Iran)	Clinic	Applied relaxation (psychoeducation and applied relaxation therapy	Usual care	(a) Birth weight (b) Preterm birth (c) Surgical delivery rate	(a) Perceived stress (b) State/Trait Anxiety (in the prenatal period)	Treatment (elevated symptoms of anxiety)	23.8 (range: not reported)	Group face-to-face plus pamphlet	Nurses	7
Brugha et al (2000)^41^	209	HIC (UK)	Clinic	Preparing for Parenthood (psychoeducation plus social support, cognitive and problem-solving skills)	Usual care	Postnatal depression (GHQ-D, EPDS, SCAN)	(a) Levels of social support (b) Problem-solving style	Indicated prevention (elevated depression symptoms)	19 (16–38)	Group face-to-face	(a) Nurses (b) Occupational therapist	7
Dugravier et al (2013)^42^	367	HIC (France)	Home	CAPEDP (psychoeducation plus parenting and social support)	Usual care	Postnatal depression (EPDS)	(a) Child psychopathology (b) Quality of home environment	Universal/ primary prevention (first time mother <26 years of age)	22.3 (range: not reported)	Individual face-to-face	Psychologists	14
Ginsburg et al (2012)^43^	47	HIC (USA)	Home	Living in Harmony (psychoeducation plus cognitive–behaviour therapy)	Active (education support)	Postnatal depression (CES-D, EPDS, DISC)	(a) Social support (b) Global functioning and support	Indicated prevention (elevated depression symptoms: EPDS > 16	18.15 (15–21)	Individual face-to-face	Family health educators	11
Kariuki et al (2021)^44^	567	LMIC (Kenya)	Clinic	Psychoeducation	Usual care	Postnatal depression (BDI)	Not applicable	Universal/ Primary prevention (mothers 6–10 weeks postpartum, aged >18 years)	52.9% aged 25 years and below	Individual face-to-face	Nurses	1
Phipps et al (2013)^45^	106	HIC (USA)	Clinic	REACH (psychoeducation plus interpersonal therapy – developing healthy nurturing relationships)	Active (book on baby basics)	Postnatal depression (KID-SCID)	Not applicable	Universal/primary prevention (age <17 years when they conceived)	16 (13–18)	Individual face-to-face and group face-to-face		6
Sangsawang et al (2021)^46^	40	UMIC (Thailand)	(a) Clinic (b) Home	Midwife-family provided social support (MFPSS) programme (psychoeducation plus social support)	Usual care	Postnatal depression (EPDS)	Rate (percentage with EPDS score ≥ 13) and severity of postnatal depression	Universal/primary prevention (first time mothers aged 10–19 years)	17.4 (10–19)	(a) Individual face-to-face (b) Telephone contact	Midwives	8
Zlotnick et al (2006)^47^	99	HIC (USA)	Clinic	The ROSE Programme (psychoeducation plus interpersonal therapy	Usual care	Postnatal depression (BDI)	(a) Depression severity (b) Social adjustment	Indicated prevention (risk of PPD as measured by Cooper's Risk Survey)	22.4 (range: not reported)	Group face-to-face	Nurses	5
**Qualitative studies**												
Abrams et al (2016)^48^	*N* = 14; 86% pregnant	Vietnam	Community, health centres	Semi-structured interviews; perceptions and experiences of perinatal mental disorders – depression and anxiety	Not applicable	Not applicable	Not applicable	Not applicable	Median = 25 (21–36)	Not applicable	Not applicable	Not applicable
Baldisserotto et al (2020)^49^	*N* = 26; 69% pregnant	Brazil	Community, primary healthcare units	Focus groups; barriers to seeking and accepting treatment for perinatal depression	Not applicable	Not applicable	Not applicable	Not applicable	24.9 (range: not reported)	Not applicable	Not applicable	Not applicable
Boath et al (2013)^50^	*N* = 15; all postpartum	UK	Community, primary care trusts	Semi-structured interviews; experiences of being a teenage mother; key support needs for provision by formal and informal support; and the potential for support and education to be delivered by healthcare workers or peers	Not applicable	Not applicable	Not applicable	Not applicable	18.8 (17–19)	Not applicable	Not applicable	Not applicable
Cadigan & Skinner (2015)^51^	*N* = 32; all pregnant at baseline assessment	USA	Community, county health departments, clinics of nutrition programme, parenting classes, and local maternity clinics and fairs	Longitudinal ethnographic Interviews; beliefs and experiences related to depression, including perceptions of its cause, symptoms, trajectory or prognosis, appropriate treatment and management, and its emotional and social impact (e.g. stigma)	Not applicable	Not applicable	Not applicable	Not applicable	(Range: 16–40, with 17 perinatal females (53%) aged 25 years or under)	Not applicable	Not applicable	Not applicable
Guy et al (2014)^52^	*N* = 25; all postpartum	USA	Community	Focus groups: mental health literacy in postpartum period	Not applicable	Not applicable	Not applicable	Not applicable	24.3 (range: not reported)	Not applicable	Not applicable	Not applicable
Kinser & Masho (2015)^53^	*N* = 17; all pregnant	USA	Community	Focus groups; experience of stress and depression in pregnancy and perceptions of adjunctive non-pharmacologic management strategies, such as mind–body therapies and other prenatal activities	Not applicable	Not applicable	Not applicable	Not applicable	17.5 (14–21)	Not applicable	Not applicable	Not applicable
Kola et al (2020)^54^	*N* = 17; all postpartum	Nigeria	Community	Focus groups; views on the factors that promote or hinder help-seeking and engagement	Not applicable	Not applicable	Not applicable	Not applicable	22 (range: not reported)	Not applicable	Not applicable	Not applicable
Logsdon et al (2009)^55^	*N* = 9; all postpartum	USA	Community; teen parent programme that is an option of the public school system	Focus groups; knowledge and perceptions about the symptoms of depression and the barriers to depression treatment	Not applicable	Not applicable	Not applicable	Not applicable	16 (13–17)	Not applicable	Not applicable	Not applicable
Logsdon et al (2010)^56^	*N* = 5; 0% pregnant	USA	Community	Semi-structured interviews; satisfaction with their experience in the study that uses an education component (education about symptoms of depression and the importance of evaluation and treatment)	Not applicable	Not applicable	Not applicable	Not applicable	Mean age: not reported (13–18)	Not applicable	Not applicable	Not applicable
Nakku et al (2016)^57^	*N* = 48; 50% pregnant	Uganda	Community, maternity clinic of the district hospital	Focus groups; barriers and facilitators, as well as perceptions about the feasibility and acceptability of plans to deliver perinatal mental healthcare in primary care settings in a low-income, rural district	Not applicable	Not applicable	Not applicable	Not applicable	Pregnant: 22.8 (18–33); postpartum: 29.83 (22–42)	Not applicable	Not applicable	Not applicable
Recto & Champion (2018)^58^	*N* = 20; 40% pregnant	USA	Community, parenting classes across urban high schools	Semi-structured interviews; recognising depression, perceptions of professional help and attitudes concerning perinatal depression	Not applicable	Not applicable	Not applicable	Not applicable	17.2 (15–19)	Not applicable	Not applicable	Not applicable
Recto & Champion (2018)^59^	*N* = 20, 40% pregnant	USA	Community, parenting classes across urban high schools	Semi-structured interviews; knowledge and beliefs concerning perinatal depression	Not applicable	Not applicable	Not applicable	Not applicable	17.2 (15–19)	Not applicable	Not applicable	Not applicable

Method refers to the intervention description for quantitative studies, and data collection method for qualitative studies. UMIC, upper-middle-income country; HIC, high-income country; GHQ-D, General Health Questionnaire; EPDS, Edinburgh Postnatal Depression Scale; SCAN, Schedules for Clinical Assessment in Neuropsychiatry; CAPEDP, Parental Skills and Attachment in Early Childhood: Reducing Mental Health Risks and Promoting Resilience; CES-D, Centre for Epidemiologic Studies Depression Scale; DISC, Diagnostic Interview Schedule for Children; LMIC, low- or middle-income country; BDI, Beck Depression Inventory; REACH, Relaxation, Encouragement, Appreciation, Communication, Helpfulness; KID-SCID, Structured Clinical Interview for the DSM-IV Childhood Diagnoses; ROSE, Reach Out, Stand Strong, Essentials for New Mothers; PPD, postpartum depression.

Eight of the eligible studies were quantitative,^40–47^ and examined interventions containing psychoeducation addressing perinatal depression or anxiety. Studies were conducted in the USA (*n* = 3), France (*n* = 1), Iran (*n* = 1), Kenya (*n* = 1), Thailand (*n* = 1) and the UK (*n* = 1). Sample sizes varied from 40 to 567 participants; all participants identified as female. Seven of the quantitative studies utilised a randomised controlled trial design, apart from one, which was longitudinal quasi-experimental (participants were not randomly assigned to the experimental or control group).^44^ Seven studies focused on perinatal depression and only one^40^ focused on anxiety. Six interventions were delivered in the prenatal period with outcomes measured postnatally. In addition, one intervention was delivered postnatally,^44^ and one was delivered prenatally with outcomes measured during the prenatal period.^38^ Outcomes were measured in terms of diagnosis (*n* = 1), symptoms (*n* = 6) or both (*n* = 1).

Twelve qualitative studies were deemed eligible.^48–59^ The studies were conducted in the USA (*n* = 7), UK (*n* = 1), Brazil (*n* = 1), Vietnam (*n* = 1), Uganda (*n* = 1) and Nigeria (*n* = 1). Data collection methods included focus groups (*n* = 6), semi-structured individual interviews (*n* = 5) and longitudinal ethnographic interviews (*n* = 1).

**Fig. 2:**
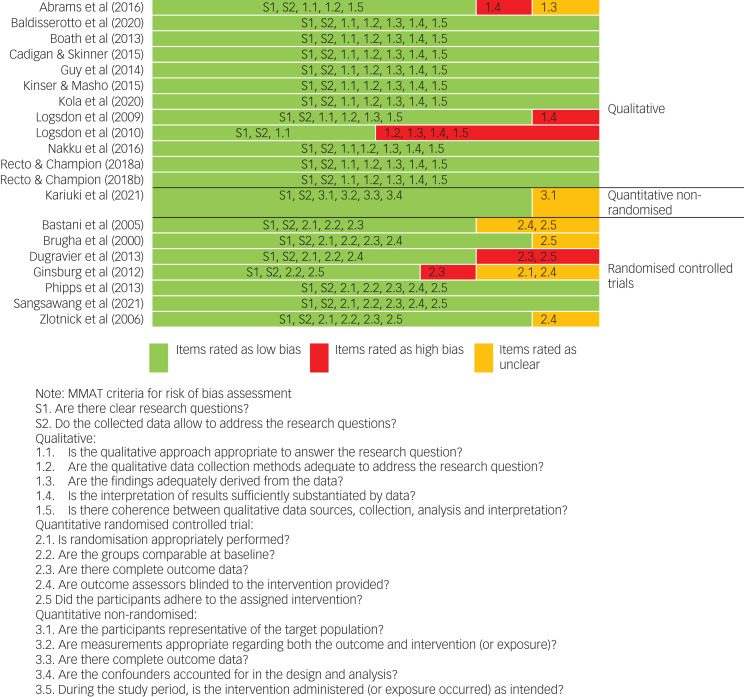
Quality assessment of the included studies. For studies Recto & Champion, 2018a refers to reference 58 and 2018b refers to reference 59. MMAT, Mixed Method Assessment Tool.

Around half (55%) of the included studies were rated as high quality (Fig. 2); 40% of the included studies were considered as having a moderate risk of bias and the remainder (10%) were rated as having high risk of bias overall. This implies that most of the included studies indicate the true treatment effects with relatively low risk of bias.^60^ The individual items that were most commonly rated as having high risk of bias were incomplete outcome data (for quantitative studies), and interpretation of results not being adequately supported by the data, evidenced by an absence of quotes from interview participants (for qualitative studies). Items most commonly rated as unclear were blinding of assessors and attrition rates.

### Evidence synthesis

#### Research question 1: Does psychoeducation work and for whom?

A summary of quantitative results is presented in Table 2. Seven of the included studies reported on depression outcomes. One study^44^ reported on a standalone psychoeducation intervention for the primary prevention of PPD in new mothers (52.9% were aged under 25 years). The study found that psychoeducation significantly reduced depressive symptoms in the intervention group (*P* < 0.001) as compared with the control (usual care) group (*P* = 0.71), with between-group differences indicating moderate effects (*d* = 0.32). Six studies^41–43,45–47^ examined multicomponent interventions with psychoeducation for preventing PPD. Three^41,44,45^ focused on universal/primary prevention (participants were not included based on diagnosis or elevated symptoms of depression) and three^41,43,47^ focused on indicated prevention (participants recruited based on elevated symptoms of depression or risk of PPD). One study^46^ reported a significant effect of the intervention, compared with usual care on preventing PPD in first-time adolescent mothers with a mean age of 17.4 years, with large interventional effects (*P* < 0.05, *d* = 1.73). Zlotnick et al^47^ studied a sample of 99 pregnant women (mean age 22.4 years). It was found that the multicomponent intervention with psychoeducation reduced incidence of PPD at 3 months postpartum (4 and 20% of participants developed PPD at 3 months postpartum in the intervention and usual care (control) groups, respectively; *P* = 0.04). However, there was no effect of the intervention on severity of depressive symptoms. A study by Phipps et al^45^ showed a lower incidence rate of PPD at 6 months postpartum for the intervention group (12.5%) when compared against the active control group (received a comprehensive pregnancy guide only), in a sample of 106 pregnant adolescents aged 13–18 years. However, the small sample size (*n* = 106) was underpowered to detect a statistically significant effect for the intervention. Ginsburg et al^43^ found similar pre–post reductions in depression symptoms within intervention and control (education support – not focusing on mental health) groups delivered for a sample of pregnant young mothers aged 15–21 years (mean age 18.2 years). Study authors attributed this non-significant between-group difference to the study being underpowered (*n* = 47); non-specific therapeutic effects, since both groups received a weekly visit from an interventionist; and increased optimism, knowledge and self-efficacy as a result of providing information about pregnancy to participants in the control group.

**Table 2:** Summary of quantitative evidence

Study	Study type	Outcome	Outcome measure	Intervention	Study *N*	Baseline mean (s.d.)	Follow-up (postpartum) mean (s.d.)	*P-*value	Cohen's *d* (95% CI)
Bastani et al^a^ (2005)^40^	RCT with a prospective experimental design	State/trait anxiety	STAI	Intervention	55	Not applicable	Not applicable	*P* < 0.001	Not applicable
				Control	55	Not applicable	Not applicable		
Brugha et al^b^ (2000)^41^	RCT	Depression	GHQ-D	Intervention	24	Not applicable	Odds ratio 1.22	*P* = 0.55	*d*: Not applicable (0.63–2.39)
				Control	21	Not applicable			
			EPDS	Intervention	15	Not applicable	Odds ratio 0.82	*P* = 0.61	*d*: Not applicable (0.39–1.75)
				Control	18	Not applicable			
			SCAN	Intervention	3	Not applicable	Odds ratio 0.48	*P* = 0.30	*d*: Not applicable (0.12–1.99)
				Control	6	Not applicable			
Dugravier et al (2013)^42^	RCT	Depression	EPDS	Intervention	183	10.5(5.6)	8.6 (5.4)	*P* = 0.28	0.04 (0.35–1.34)
				Control	184	11.1(5.6)	9.4 (5.4)	*P* = 0.18 (adjusted *P* = 0.33)	
Ginsburg et al^c^ (2012)^43^	RCT	Depression	CES-D	Intervention	22	22.00 (8.28)	11.42 (3.60)	Not applicable	0.05 (CI: not applicable)
				Control	25	21.44 (7.38)	11.45 (7.24)	Not applicable	
			EPDS	Intervention	22	9.32 (5.96)	8.50 (3.85)	Not applicable	0.11 (CI: not applicable)
				Control	25	8.44 (5.76)	7.66 (4.22)	Not applicable	
Kariuki et al (2021)^44^	Longitudinal quasi-experimental design (non-randomised)	Depression	BDI	Intervention	284	8.08 (9.83)	5.20 (7.93)	*P* < 0.001	0.32 (−3.06 to −0.07)
				Control	283	6.98(7.49)	6.82 (8.34)	*P* = 0.708	
Phipps et al^d^ (2013)^45^	RCT	Depression	KID-SCID	Intervention	54	Not applicable	*n*/*N* (%) 6/48 (12.5)	Not applicable	*d*: Not applicable (3.1–21.9)
				Control	52	Not applicable	*n*/*N* (%) 13/52 (25.0)	Not applicable	*d*: Not applicable (13.2–36.8)
Sangsawang et al (2021)^46^	RCT	Depression	EPDS	Intervention	20	7.1	5.25	*P* < 0.05	1.73 (CI: not applicable)
				Control	20	7.0	11.1	*P* < 0.001	
Zlotnick et al^e^ (2006)^47^	RCT	Depression	BDI	Intervention	53	15.3 (6.96)	9.39 (7.42)	Not applicable	0.001 (CI: not applicable)
				Control	46	16.0 (7.77)	10.1(9.41)	Not applicable	

RCT, randomised controlled trial; STAI, State-Trait Anxiety Inventory; GHQ-D, General Health Questionnaire; EPDS, Edinburgh Postnatal Depression Scale; SCAN, Schedules for Clinical Assessment in Neuropsychiatry; CES-D, Centre for Epidemiologic Studies Depression Scale; BDI, Beck Depression Inventory; KID-SCID, Structured Clinical Interview for the DSM-IV Childhood Diagnoses.

a. No means were reported. However, *P*-values for between-group analyses were reported: there were no significant differences in state/trait anxiety (*P* =0.332, *P* = 0.052)

between the groups at baseline. At post-intervention, scores for state/trait anxiety showed significant decreases in the intervention group when compared with the control group (*P* < 0.001).

b. Odds of being a case of postnatal depression were reported only for participants who completed a sufficient number of sessions. Confidence intervals were reported but effect sizes were not reported. There was insufficient information to calculate effect sizes.

c. Exact *P*-values were not reported; however, it was reported that there were no significant differences between groups for each outcome measure. Effect sizes (Cohen's *d*) have been reported. The intervention showed potential in reducing depressive symptoms. Results were comparable at all follow-ups. The study also measured onset of major depressive disorder using the Diagnostic Interview Schedule for Children (DISC). There were no participants with a diagnosis of major depressive disorder at baseline, only two (8%) participants in the education support (control) group developed major depressive disorder across all assessment points.

d. No baseline characteristics were reported. Incidence of major depression (i.e. number of cases for each group) at follow-up 1 and complete follow-up (in the postnatal period) was reported. The results were comparable at both follow-ups. Confidence intervals were reported but effect sizes were not reported. There was insufficient information to calculate effect sizes.

e. *P*-values were not reported. However, incidence was reported: two (4%) participants in the intervention group and eight (20%) participants in the control group developed/were diagnosed with depression at 3 months postpartum (*P* = 0.04).

Null effects were also reported by Brugha et al^41^ when comparing intervention with the usual care (control) group in a sample of 209 pregnant women (mean age 19.0 years). Similarly, a study by Dugravier et al^42^ did not find an effect of the multicomponent intervention with psychoeducation on PPD (*P* = 0.28) when delivered for a sample of 327 pregnant women (mean age 22.3 years). However, *post hoc* analysis in this study revealed that in certain subgroups, the intervention resulted in significantly lower scores: participants with fewer depressive symptoms at recruitment (*P* = 0.05), women with a partner involved in raising the child (*P* = 0.04) and women with a higher level of education (*P* = 0.05).

One study^40^ (treatment) reported on anxiety as a secondary outcome (birth outcomes were the primary study outcome) in an evaluation of relaxation therapy (which included a discussion on anxiety in pregnancy) compared with usual care in a sample of 110 pregnant women (mean age 23.8 years). This intervention significantly decreased scores for state/trait anxiety in the intervention group as compared with the control group (*P* < 0.001), but the effect size was not reported.

None of the studies carried out moderation analyses to examine the effect of age or clinical severity. Comparing across studies revealed no obvious trends. Also, the qualitative studies included in this review did not address the question related to potential moderators of effectiveness or acceptability of psychoeducation interventions, such as clinical severity, or age of young mothers.

In discussing with the YAG, the panel indicated that a key strength of psychoeducation was the focus on increasing awareness of mental health problems and preparedness to face stressors that can negatively affect mental health during pregnancy and following childbirth (Table 3). It was felt that improved mental health literacy owing to psychoeducation would be equally relevant to depression and anxiety. It was also noted that psychoeducation has potential benefits when delivered both prenatally and postnatally.

**Table 3:** Meta-synthesis of qualitative evidence and youth advisory group lived experience

Themes	Illustrative comments from qualitative studies	Illustrative comments from the youth advisory group (YAG)
Research question 1: Does psychoeducation work, and for whom?		
Limitations	Not applicable	‘[Psychoeducation] would work better with other interventions such as social support that we can get from family because I think everybody is different, and how we might handle depression matters therefore the information and skills/coping mechanisms we need also differ. It may happen that psychoeducation may be sufficient to one but may not be sufficient for another person, as such we need to combine it with other interventions for maximum benefit.’ ‘I feel like the men are also affected during the pregnancy or after birth period but tend to be neglected … They are affected in a way that they are part of the pregnancy itself in some kind of way … And they go through a lot of stress and pressure.’
Age	Not applicable	‘It would work better for prevention especially in younger adolescents.’ ‘According to the Malawian context, I think adolescents could benefit more because they are the ones who get affected by the early pregnancies which can be very traumatic because of our traditions and societies.’
Timing	Not applicable	‘Psychoeducation works best for both the antenatal or after birth but mostly after birth because you get a lot of depression and it's even more complicated when it's your first baby because you literally have no clue how to handle things.’ ‘It works better in the antenatal period because during this period one has just started going through the changes and is overwhelmed about everything that is happening around her.’
Research question 2: In which settings does psychoeducation work?		
Sources of information	Participants mentioned various sources for obtaining mental health information and did not express preference for one specific source. Online search engines and depression websites were mentioned as potential sources.^59^ Online search engines and depression websites were mentioned as potential sources.^57^ Presenting information electronically is accessible to young mothers as they do not have to leave their home and can watch it while their baby is asleep.^50^ Among formats of psychoeducation, it was noted that leaflets could be useful, but they need to be brief and targeted at young parents: ‘ … Some leaflets are just bullet pointing information. I think I would be more interested in reading about teenage mums. If you can read something quick whilst you're having a cup of tea, or doing something quick’.^50^ Also, participants expressed preferences for conversational formats rather than self-study formats. ‘I got leaflets, they were ok, but I felt better when you can talk to somebody rather than just books.’^50^ ‘I'd go to my mom or the doctor. If I had an appointment, I'd talk to them about it, and they'll most likely assist me with help. Online, looking for counselling maybe. Things that will help with the depression.’^59^	‘I would feel comfortable with a health worker since I think they wouldn't be very judging in handling my situation and also provide me with some accurate information.’ ‘The person would seem more trustworthy simply by the type of approach they use when they talk to you … An approach that is showing interest and politeness rather than a harsh and judging approach.’
Peer group formats	Group formats were valued, especially where professional health support is limited/unavailable.^50^ ‘ … and you can go in that group when you are stressed and by the time you leave the group, stress is off.’^57^ ‘You don't really know about a lot of the support networks that are there for you.’^50^ ‘[It would be good to develop] relationships with people who are going through the same thing you are, and they're your age.’^53^	‘I think the format I would prefer is a face to face and also a support group with peers who we have the same experience … Face to face is more believable and convincing.’ ‘I am part of one [support group] on Facebook that's about young mothers and it is very helpful.’ ‘I'm actually in one of the groups [for young moms] on WhatsApp … I do find the group helpful, like this other time we talked about postpartum depression and how to deal with it.’
Media formats	Media can have both positive and negative influence. ‘I was ashamed to go and say anything to the doctor and ask him about it until [my husband] saw that bipolar [commercial] and I thought wow, a lot of the symptoms apply and [the doctor] told me that I was just suffering from severe depression.’^51^ ‘Because the problems that those teen moms are having in shows or movies or just any on social media, is basically what really happens.’^59^ ‘Like I was watching a movie and this girl was depressed and like she was being really over-judged and she was all like crying really loud, like she was dying - It just turns me off more.’^59^	‘Social media would be useful and it would have a wide range of information.’ ‘Social media can have some defects as some things tend to be not true.’
Setting/location	Not applicable	‘It would work better in a clinical setting because I think I would be more confident about that with it being more professional. It should also be in a group where we would share our experiences too. But I wouldn't want a family member involved because I wouldn't be comfortable.’ ‘It would work better at home because it is convenient, and you are in a comfortable space. Your partner would also be there.’
Research question 3: Why does psychoeducation work?		
Awareness of symptoms of perinatal depression and anxiety	Currently, there is a widespread lack of knowledge about perinatal depression and anxiety, which can prevent early diagnosis and intervention. Psychoeducation can increase ability of recognising symptoms of perinatal depression and anxiety. ‘There are days when we are more introverted, sadder … but if this is depression, I do not know.’^49^ ‘I didn't really know the meaning of it [postpartum depression] really, nobody has ever told me about it.’^50^ ‘They can let you know about symptoms, what you can do. If she's feeling depressed and she doesn't know it. Maybe when she's talking about something she can realize … So having that information would be helpful.’ ^58^	‘[Psychoeducation] helps one to prepare in advance or to be aware of the harm that depression can bring. As a result, a person can prevent that from happening because of the awareness.’ ‘When someone provides you with information about stress and depression you are more aware of what you are dealing with and it is easier to prevent or get rid of.’
Awareness of self-management strategies	Participants recognised the importance of learning how to cope with stress and feelings of depression, both during pregnancy and postpartum. ‘[Intervention] gave insight to me on things that could help me that I didn't know about.’^56^	‘I read something about doing some exercises that helps to relieve some stress and keep your mind healthy.’
Awareness of formal support services	Participants commented on importance of getting clear and trustable information about relevant professional mental health services. ‘The first thing people do is go to mental health professionals and say they are depressed and start taking pills …. ’^51^	Not applicable
Dealing with fears of stigmatisation	Fears of stigma and prejudice have been reported by many participants. Fears may discourage young mothers from opening up about their feelings and may act as a barrier to the diagnosis of perinatal depression, and anxiety and prevent early intervention. They could also discourage contact with healthcare providers. These fears may result from the lack of knowledge/professional information.^49^ Thus, psychoeducation plays an important role for reducing fears of stigmatisation. ‘She probably would be judged… Maybe like they'll think she's weak, and I guess, immature.’^58^ ‘A woman with depression can't be a good mother.’^49^ ‘I had a bad experience one day with a nurse … She told me that I got pregnant when my mates were in school … I felt very worthless … ’^54^	Not applicable
Working alliance	‘I have someone to talk to and didn't have to be embarrassed because she understood what you going through because she works with people like you.’^56^ ‘I met matron [name] and she gave me hope. She told me I had a sickness of the mind that made me sad … and that I will get better with time and I did. I like her a lot.’^54^ ‘ … I was in a bad state when I went to the clinic … I thought my life was over. However, the more I visited the clinic and talked to the matron, the better I felt.’^54^	‘It validates one's feelings. When you talk to someone you actually feel good and assertive that someone actually really cares about you and your feelings.’

On the other hand, the YAG felt that psychoeducation was in itself not sufficient to address all the needs of young parents during both the prenatal and postpartum periods. This echoed the view that no single ‘ingredient’ would be able to fully address perinatal mental disorders. Therefore, psychoeducation was considered less potent as standalone programme than a multicomponent programme. Specifically, the YAG said they would benefit from lessons on transitioning to motherhood in addition to psychoeducation.

In terms of age appropriateness for perinatal psychoeducation, there was consensus that adolescents aged under 18 years would derive greatest benefit, as they may be more at risk of depression and anxiety during the perinatal period because of the stigma associated with early pregnancies. This aspect was specifically highlighted by youth advisors from Malawi, where many young women give birth before 18 years of age.

#### Research question 2: In which contexts and settings does psychoeducation work?

Five studies were conducted in high-income countries and three were completed in LMICs (Kenya, Thailand and Iran). The LMIC studies indicated that psychoeducation was effective in preventing PPD (*P* < 0.001, *d* = 0.32^44^; *P* < 0.05, *d* = 1.73)^46^ and reducing symptoms of anxiety (*P* < 0.001).^40^ Only one study from high-income countries provided evidence for the effectiveness of psychoeducation (*P* = 0.04).^47^ In terms of duration, three psychoeducation interventions were brief (one to six sessions), with two being effective (*P* < 0.001, *d* = 0.32;^44^ *P* = 0.04)^47^ and one being ineffective.^45^ Longer interventions^41–43,46^ also yielded mixed results, with two being effective (*P* < 0.001;^40^ *P* < 0.05, *d* = 1.73)^46^ and three being ineffective.^41–43^

For interventions delivered face to face, effectiveness was reported for two interventions (*P* < 0.001, *d* = 0.32;^44^ *P* < 0.05, *d* = 1.73),^46^ whereas two studies were reported to be ineffective.^42,43^ Two group interventions were found to be effective (*P* < 0.001;^40^ *P* = 0.04),^47^ whereas one was ineffective.^41^ One study delivered through a combination of one-to-one and group delivery was found to be ineffective.^45^ Out of the five interventions delivered by nurses, four were effective (*P* < 0.001;^40^ *P* < 0.001, *d* = 0.32;^44^ *P* < 0.05, *d* = 1.73;^46^ *P* = 0.04)^47^ and one was ineffective.^41^ No effectiveness of the intervention was found for studies delivered by psychologists^42^ and lay counsellors.^43^ One study that was ineffective^45^ did not state what kind of facilitators were used. In terms of study setting, effectiveness was reported for a study that used a combination of clinic and home sessions (*P* < 0.05, *d* = 1.73),^46^ whereas interventions that utilised home delivery were ineffective.^42,43^ Mixed results were reported for studies delivered in health and/or child clinics, with three studies being effective (*P* < 0.001;^40^ *P* < 0.001, *d* = 0.32;^44^ *P* = 0.04)^47^ and two being ineffective.^41,45^

Qualitative evidence revealed that psychoeducation was deemed to be more engaging by the participants when delivered in brief and visually appealing formats, such as leaflets with bullet points; when incorporating stories about real people's experiences^50^ and when facilitated by another person in a kind, non-judgemental and supportive way.^59^

^‘^ … some leaflets are just bullet pointing information. I think I would be more interested in reading about teenage mums …. If you can read something quick whilst you're having a cup of tea, or doing something quick.’^50^

The role of midwives as providers of psychoeducation was highlighted by adolescent mothers as potentially problematic, as participants recalled experiences of being judged by maternal healthcare providers in LMICs.^54^ This view was not universal, as other participants reported having positive experiences with midwives and other maternal care professionals.^54^ Participants positively commented on peers delivering the psychoeducation programme saying, ‘ … they understand more what you feel may be your problems’.^50^ The positive aspects of having access to social support groups was also acknowledged by participants in the qualitative studies.^53,57^ Therefore, having group discussions within the psychoeducation intervention could have additional benefits.

‘[It would be good to develop] relationships with people who are going through the same thing you are, and they're your age.’^53^

Finally, television and social media platforms were seen as having a potentially useful role in accessing psychoeducation, as they are easily accessible by young mothers from their homes.^51^ On the other hand, participants noted that information on social media can be problematic because it may not always be accurate.^59^

^‘^I was ashamed to go and say anything to the doctor and ask him about it until [my husband] saw that bipolar [commercial] and I thought wow, a lot of the symptoms apply and [the doctor] told me that I was just suffering from severe depression.’^51^

‘Like I was watching a movie and this girl was depressed and like she was being really over-judged and she was all like crying really loud, like she was dying – It just turns me off more.’^59^

The YAG agreed that psychoeducation can be adapted across diverse contexts. Psychologists were the most preferred provider of psychoeducation as they were perceived to be more knowledgeable about mental health issues, followed by community health workers and midwives. A number of advisors also expressed a preference for accessing psychoeducation from healthcare workers in antenatal/postnatal clinics. However, other advisors argued that interventions delivered at home would be more convenient and comfortable for young mothers. There was consensus that peers, including support groups on social media, can be an important source of psychoeducation. However, young advisors believed that trustworthy information was more likely to be delivered by health professionals, who are professionally trained and more experienced in mental health, rather than peers.

#### Research question 3: Why does psychoeducation work?

None of the included studies specifically conducted mediation analyses to test for plausible mechanisms. Experiential accounts of psychoeducation highlighted possible mechanisms for psychoeducation in four linked domains (Table 3). First, several studies demonstrated a widespread lack of knowledge about symptoms of perinatal depression and anxiety, and psychoeducation was helpful at improving self-recognition of these conditions.^49,50,52,57^

^‘^I didn't really know the meaning of it [postpartum depression] really, nobody has ever told me about it. Nobody has ever told me what it is really …. ‘I just sit here sometimes and I am crying for no reason, but I could have detected it earlier if someone had explained to me what your first symptoms were, but nobody told me.’^50^

Second, psychoeducational approaches helped young mothers to learn self-management strategies for coping with stressful situations and symptoms of depression, both during pregnancy and postpartum.

‘[Intervention] gave insight to me on things that could help me that I didn't know about.’^56^

Third, information about relevant professional mental health services was also valued.^51^ Fourth, qualitative studies showed how fears of stigma (‘She probably would be judged … Maybe like they'll think she's weak, and I guess, immature’),^58^ mostly resulting from lack of professional information about the illness, can discourage young parents from opening up about their mental health difficulties. Such stigma may discourage appropriate help-seeking from healthcare providers, delaying diagnosis and treatment.^50,54^ Psychoeducation was considered effective at addressing the knowledge deficits that underpin mental health stigma, thereby encouraging formal help-seeking at an earlier stage.^49^ Relational aspects of psychoeducation were also discussed. There were indications that the positive experience of the therapeutic alliance with providers of psychoeducation led to increased motivation and hopefulness about managing future challenges.^54,56^

‘I met matron [name] and she gave me hope. She told me I had a sickness of the mind that made me sad – and that I will get better with time and I did. I like her a lot.’^54^

The youth advisors reiterated the beneficial effects of psychoeducation on coping. They stated that psychoeducation can improve self-efficacy, leading to positive expectations about the ability to deal with mental health problems in the present and future. In this way, psychoeducation was seen as a way to empower young parents and motivate further positive actions, such as seeking professional help and advice. There was also a unanimous view that the working relationship (therapeutic alliance) between the therapist and the client was a key factor as it validates one's feelings, thereby increasing the patient's adherence to the prevention or treatment plan.

## Discussion

The current review examined multiple sources of evidence for psychoeducation as a potential active ingredient in the interventions for youth perinatal depression and anxiety. There was limited evidence on the effectiveness of psychoeducation as a standalone intervention for depression, and no studies have examined anxiety outcomes resulting from psychoeducation alone. Hence, no firm conclusions about the role of psychoeducation as a standalone intervention can be drawn. However, psychoeducation was also included in effective multicomponent interventions for both depression and anxiety. Most of the evaluations considered psychoeducation interventions delivered during the prenatal period, with only one study considering a prenatal intervention. Moreover, all but one study considered preventive intervention (i.e. interventions were delivered universally during the prenatal period and outcomes were measured postnatally). However, insights from the YAG suggested that psychoeducation could have potential benefits when delivered both prenatally and postnatally. There were also indications that psychoeducation interventions are generalisable across diverse contexts, with the strongest evidence emerging for studies conducted in LMICs. Several of the included studies conducted in high-income countries did not demonstrate significant effects.

The three quantitative studies which focused specifically on adolescents and youth within the 14–24 year age range showed mixed results. One multicomponent intervention with psychoeducation^46^ was found to be effective in preventing PPD in first time adolescent mothers. However, two studies showed no evidence for the effectiveness of psychoeducation: one study^43^ showed similar reduction in symptoms from pre–post between the intervention and control groups; the other study^45^ showed only post-intervention scores in each group, with no indication of baseline scores or relative improvements. Although a lower incidence rate of PPD was reported for the intervention group as compared with the control group at follow-up, this change was not statistically significant.

Although child outcomes were not reported in the quantitative studies included in this review, other research suggests a strong link between maternal mental health outcomes and child outcomes. Studies^61,62^ have found a bi-directional effect of multicomponent interventions with psychoeducation that target the mother–infant dyad on emotional and behavioural outcomes. This implies that integrating psychoeducation with content that covers prenatal and postnatal topics (e.g. knowledge and skills related to pregnancy, child-rearing and early child development) could be effective in addressing perinatal depression and anxiety. This was also reiterated by the YAG, who said they would benefit from lessons on transitioning to motherhood in addition to psychoeducation.

Although the quantitative studies showed that most of the interventions were delivered by nurses/midwives, findings from qualitative studies revealed that engaging midwives in youth maternal-based interventions could be a challenge if they express judgement of adolescent pregnancy. Other research has shown that implementation of task-sharing interventions (i.e. where non-mental-health specialists deliver psychological interventions) may be impeded in situations where busy health workers have to take on extra tasks, training and supervision alongside their normal roles.^63^ In line with the association between therapeutic alliance and mental health outcomes in young people,^64^ a supportive, kind and non-judgemental therapist was identified as being a key facilitator in the delivery of psychoeducational interventions. Although there has been substantial attention given to the potential public health benefits of digital interventions and the use of self-directed (online) modes to reduce human resources and increase access to mental health at a wider population level (although none of our included studies looked at digital delivery), research has shown that some degree of human facilitation tends to increase engagement with and adherence to digital interventions.^65^ Considering how negatively social isolation can affect women during pregnancy and the postpartum, this relational aspect may be particularly salient for young women in the perinatal period, who may struggle to find time and motivation for self-directed skill building.^66^

Commentaries from the YAG suggest that although psychoeducation interventions can target both adolescents and young adults, younger adolescents may benefit more because being a young mother can be more challenging. In addition, society tends to frown more on teenage pregnancies. This is in line with studies that show that young age of the mother is a common risk factor for perinatal depression and anxiety in adolescents, as they have to adapt to the new role alongside dealing with their own developmental changes.^67,68^ In addition, studies have shown that younger adolescents are at higher risk of poor mental health outcomes because they are more susceptible to the effects of stigmatisation.^10^

Among possible mechanisms of actions, evidence suggested that psychoeducation can affect anxiety and depression by stimulating help-seeking (through effects on knowledge deficits that may underpin barriers such as stigma and failure to recognise symptoms), and developing adaptive skills for self-management of stressors and symptoms (with concomitant effects on secondary appraisals of coping ability). Intervention engagement and motivation for behaviour change can be enhanced through a positive working alliance with providers of psychoeducation. This is in line with the World Health Organization guidelines on the integration of perinatal mental health in maternal and child health services,^69^ which state that providing psychoeducation to perinatal women and their partners or family members in supportive environments can help increase awareness of symptoms and knowledge options, reduce stigma and enhance coping.

### Strengths and limitations

This is the first review of evidence specific to psychoeducation interventions for perinatal depression and anxiety in youth. In this review, we looked at diverse evidence from both quantitative and qualitative studies, complemented with commentaries from the international YAG. This provided further depth and credibility to our findings. Nevertheless, the review is based on findings from studies published in English and peer-reviewed studies only. However, not including unpublished or non-peer-reviewed findings, and not contacting authors for missing information, may lead to a potential bias in favour of an effect. Because of limited time and resources, only 10% of abstracts and full texts were double-screened with a kappa value of 0.75, which is less than the recommended value of 0.81 and above. This means that some relevant studies may have been missed by independent reviewers. However, researchers^35,70^ have argued that kappa values between 0.61 and 0.80 indicate substantial interrater reliability. Additionally, search terms were limited to title and abstract only. Therefore, it is likely that some relevant studies might have been missed, as most multicomponent interventions may not explicitly specify ‘education’ or related terms in title or abstract, even if these are included in the intervention package. Another limitation of the study is that none of the included studies included measures of mental health literacy to determine whether the decrease in symptoms of depression and anxiety could be attributed specifically to psychoeducation. However, YAG insights were sought on the importance of psychoeducation. The review focused on mothers aged 14–24 years. However, studies where the mean participant age was less than 25 years or if 50% of the sample were under 25 years were included. Therefore, only three out of eight quantitative studies, and six out of 12 qualitative studies, were restricted to participants in the target age range. All other studies had older participants or insufficient information to determine age range. However, Lieberman et al^26^ suggested that adapting interventions that successfully address depression and anxiety in older perinatal women could be useful for researchers targeting perinatal depression and anxiety in youth. Moreover, most studies included a majority of participants from the target age range, with the mean participant age being under 25 years.

In conclusion, psychoeducation has been used as a practice element in interventions for perinatal depression and anxiety in various contexts and across different age ranges. The current review found limited evidence for psychoeducation as a standalone intervention and mixed results for the effectiveness of psychoeducation in multicomponent interventions. Nevertheless, there was some evidence that psychoeducation holds promise in addressing perinatal depression and potentially perinatal anxiety in youth, when offered in combination with other elements. Because of its flexibility and simplicity, psychoeducation can be adapted across various populations and settings. Although no studies included measures of mental health literacy to determine the direct effect of psychoeducation, it was a common intervention component in multicomponent interventions, some of which were effective. In addition, the usefulness of psychoeducation was endorsed by the YAG. Therefore, psychoeducation, especially when offered as part of multicomponent interventions, could be a key foundational ingredient for promoting positive outcomes among youth with perinatal depression and/or anxiety, as it animates help-seeking and self-care. To conclude, findings from this review can be used to improve and strengthen interventions offered to young parents (Box 2). It is critical that healthcare providers, communities and researchers focus on the multiple needs of this vulnerable population, particularly when designing intervention strategies.

Box 2 Directions for future research

- Many included studies are based on small samples and are likely to be underpowered to detect a significant effect, more large-scale studies are needed to test psychoeducational interventions in adequately powered trials.
- There is a lack of studies measuring mediators and moderators of psychoeducation interventions. Future studies need to focus more on exploring potential pathways and mechanisms in a sufficiently powered culturally diverse samples.
- Future studies need to consider inclusion of young fathers in psychoeducation interventions as they are also at heightened risk of mental health problems, which could consequently negatively affect their relationships with their partners and children.
- It is important to explore novel and innovative approaches to address psychoeducational needs of new generations of young parents, including online communities and peer-delivered formats.
- Involving young people with lived experience of youth pregnancy/parenthood and perinatal depression/anxiety in development of these interventions is a key.
- Researchers must consider involving adolescents and young women in the development of interventions targeting youth, to address the unique needs of this population.

## Data Availability

Data availability is not applicable to this article as no new data were created or analysed in this study.
